# Dear supplier, how sustainable are you? A multiple-case study analysis of a widespread tool for sustainable supply chain management

**DOI:** 10.1007/s00550-020-00507-z

**Published:** 2020-11-13

**Authors:** Iain J. Fraser, Martin Müller, Julia Schwarzkopf

**Affiliations:** 1 Institute of Sustainable Corporate Management, Ulm University, Helmholtzstraße 18, 89081 Ulm, Baden-Wuerttemberg Germany; 2 HTW Berlin Business School, Treskowallee 8, 10318 Berlin, Germany

**Keywords:** Sustainable supply chain management, Supplier assessment, Self-assessment, Sustainability assessment tools, Supplier assessment fatigue, Social desirability bias

## Abstract

This article analyses one of the most common tools employed by global focal companies in sustainable supply chain management (SSCM) across all industries: supplier sustainability self-assessment questionnaires. Extant research has moved beyond the questions of whether and which suppliers should be assessed. Current research is already focussing on how to share and standardise such assessment data. Despite mounting general research on SSCM, we identified that specific tools such as self-assessment questionnaires have not been empirically analysed in SSCM literature. Thus, this paper addresses the research questions of what differences there are among supplier self-assessment questionnaires and how supplier responses to such questionnaires might be influenced. Our research involves an abductive multiple-case study design and an analysis of over 25,000 responses from globally dispersed suppliers to two types of supplier sustainability self-assessment questionnaires administered and requested by a global automotive focal company.

Although the two questionnaires covered similar areas of sustainability practices and were administered to suppliers of the same focal company, the suppliers’ responses demonstrated various observable differences in average sustainability scores.

Social desirability bias and supplier assessment fatigue were identified as issues confronting such questionnaires. We find that questionnaire design, how the questionnaire is embedded in the focal company’s processes and institutional settings are factors that potentially influence suppliers’ responses and could counteract social desirability bias and supplier assessment fatigue. Based on these findings we make suggestions for improving these SSCM tools and provide recommendations for further research.

## Introduction

Two decades ago, Crane (1999) titled an article “Are you ethical? Please tick yes or no.” Over 20 years later, the title is relevant to sustainable supply chain management (SSCM) research and warrants specific academic attention. Global buying companies increasingly monitor and/or evaluate the sustainability practises of their suppliers with the help of self-assessment questionnaires but these central tools have not been featured in SSCM investigations. This is a problem because, as Das (2017) argues the challenge for SSCM as an academic discipline is “how to make the broad concepts of sustainability relevant, applicable and *operationalisable* to SCM at firm level” (Das 2017, p. 1345). The problem is, how sure are we about the accuracy of such tools for conveying suppliers’ sustainability practices and performance? And how can companies trust the responses and data generated?

The last decade has seen a veritable boom in publications focussing on SSCM, the closely-related areas of responsible/green/sustainable purchasing and supplier management (Ahi and Searcy 2013; Ansari and Kant 2017; Beske-Janssen et al. 2015; Brandenburg et al. 2019; Gimenez and Tachizawa 2012; de Oliveira et al. 2018; Seuring and Müller 2008; Koberg and Longoni 2019; Walker et al. 2012). This intensification of research corresponds with a continual increase in the importance of SSCM to practitioners/companies, as evidenced by the growth in activity within and among firms regarding sustainability in supply chains (Gimenez and Tachizawa 2012; Schoeggl et al. 2016a; Sancha et al. 2016; Singh and Trivedi 2016). At the same time we note an intensification of stakeholder expectations (Seuring and Müller 2008; Li et al. 2014; Mueller and Bessas 2017) and a densification of guiding frameworks (Rasche and Gilbert 2012; Seuring and Gold 2013; Lee and Kashmanian 2013). By now, large multi-national corporations will inevitably include their approaches to supply chain sustainability in their sustainability reporting (Harms et al. 2011; Walker and Jones 2012). Nonetheless, there still appears to be a gap between theoretical academic SSCM research on the one hand, and SSCM practice on the other. Companies confront increasing expectations of their responsibility and have to devise strategies, measures, processes and tools to improve sustainability in their supply networks as part of their broader sustainability strategies (Canzaniello et al. 2017; Ecovadis 2017; Pagell and Shevchenko 2014).

**Fig. 1:**
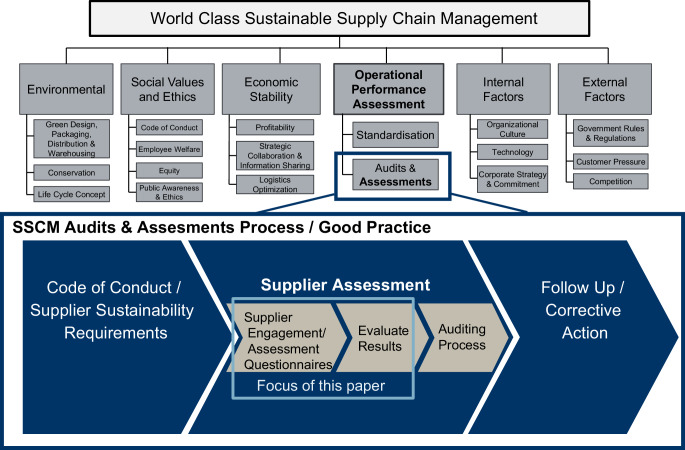
Position of our research in existing frameworks. (Authors’ own depiction, adapted—with permission—from Dubey et al. (2017a) and Mueller and Bessas (2017))

Various practical approaches and tools employed by multi-national corporations to improve sustainability in their supply chains have been identified (Lee and Kashmanian 2013). Whilst there has been illuminating analysis of the effectiveness of codes of conduct (Egels-Zandén 2014; Bartley and Egels-Zandén 2015) and to a lesser extent audits (Locke 2013; Short et al. 2016; Terwindt and Armstrong 2019), there has otherwise been little focus in SSCM literature on self-assessment as a tool for SSCM. One example is provided by Kashmanian and Moore (2014), whose research primarily focussed on company codes of conduct, which follow recommendations to companies by the United Nations Global Compact (UNGC) and Business for Social Responsibility (BSR). In summarising supplier monitoring activity, they briefly mention supplier self-assessments: “Prior to conducting audits of any kind, a company usually has suppliers perform self-assessments”, which they point out are cheaper and faster in terms of identifying sustainability risk (Kashmanian and Moore 2014, pp. 13–14). Das’ (2017) review of the state of SSCM research included a framework for SSCM practices, however supplier questionnaires (or similar SSCM performance measurement tools) were not covered. Thus, in this regard it appears that SSCM as an academic discipline is still challenged to be managerially relevant (Carter and Easton 2011) in the sense that it has not analysed the processes and tools that are widely in use. This is supported by Dubey et al. (2017a) who suggest a framework for bundling the numerous theoretical and practical oriented academic works in the field of SSCM (see Fig. 1). One of their constructs, “Operational Performance Assessment” and the corresponding item “Audit and Assessment” (Dubey et al. 2017a, p. 339), forms the particular frame for the current paper. The tools covered by their framework are central to much focal company SSCM work, employed in multiple industries, and are sometimes the sole source of supply chain sustainability information. However, we argue that questionnaires are often taken for granted or overlooked and thus we find that they “must be further scrutinized through phenomenon-driven research” (Hahn and Ince 2016, p. 34).

This paper aims to partially address this research gap, understanding supplier sustainability questionnaires to be part of a supplier assessment process (Mueller and Bessas 2017) and thus it focuses on the following research questions:

RQ1: What are the differences between an in-house, one-off supplier self-assessment questionnaire and an industry-wide, shared and validated supplier self-assessment?

RQ2: What might the major factors influencing supplier responses to these two types of sustainability self-assessment questionnaires be?

By answering these research questions with empirical case data on these central tools for SSCM, our research we postulate various factors that may explain substantial differences in the results of suppliers’ sustainability self-assessment questionnaires and make suggestions about how to improve the quality of such tools in the future. Following this introduction, we now provide a literature review of supplier assessment in SSCM, supplier self-assessment and challenges to self-assessment questionnaires, such as social desirability bias. Thereafter, we introduce the multiple-case study methodology and focus on supplier sustainability questionnaires as the unit of analysis for cross-case synthesis. We then present the case studies, describing how the data was collected and analysed. Then the results of the cases are presented, compared and synthesised. After this comparative analysis and discussion of both cases, we present our conclusions, including the implications of our findings, limitations of the research and recommendations to both practitioners and academics for areas of improvement and future research.

## Literature review

Global companies have been profiting from globally dispersed supplier networks for many decades (Gunasekaran et al. 2004; Lebaron et al. 2017; Lee and Kashmanian 2013; Petersen et al. 2000). Since the 1990s, public attention has increasingly been drawn to the negative effects of companies’ practices and the perception of their responsibility for sustainability problems in global supply chains (Caniëls et al. 2013; Foerstl et al. 2010; Frostenson and Prenkert 2015; Klein 2010; Locke 2013; Seuring and Müller 2008; Wolf 2014). The emergence of SSCM can be seen as a response to this stakeholder pressure (Seuring and Müller 2008). Beske-Janssen et al. (2015) documented the remarkable growth of academic publications on the topic of SSCM. Ansari and Kant (2017) hail SSCM’s rise to prominence as the “advent of a new era” (ibid, p. 2524). Most SSCM literature has an implicit or explicit focus on so-called focal companies (Frostenson and Prenkert 2015), which typically exhibit the following characteristics: “(1) rule or govern the supply chain, (2) provide the direct contact to the customer, and (3) design the product or service offered” (Seuring and Müller 2008, p. 1699) and tend to be the owners/drivers of supplier sustainability assessments in their respective supply chains.

### SSCM assessment

The SSCM tools and processes that focal companies have at their disposal are applicable across industries (Hoejmose et al. 2013; BSR & UNGC 2015; Mueller and Bessas 2017). For example, Schoeggl et al. (2016b) note that companies in both the electronics and automotive industries are trying to address myriad sustainability issues in their broad and overlapping supply chains through sustainability assessment. As focal companies are increasingly required to assess their supply chains for sustainability risks (Lechler et al. 2019), they must first understand the status quo of SSCM in their supply chain, before they can improve it. To achieve this, they must be able to gauge progress in their suppliers’ sustainability performance, which means supplier sustainability performance must be measurable and then assessed (Mokhtar et al. 2017; Reilly 2017). Thus, as Seuring and Gold (2013, p. 2) find, “managing the link to suppliers plays a key role when focal firms aim at moving toward sustainability.”

Spence and Bourlakis (2009, p. 27) and Foerstl et al. (2010) have argued that supplier assessment has a positive effect on sustainability performance. Mueller (2017) proposes companies break down their SSCM processes into three steps: prevention, early warning, and reaction (see Fig. 1). Companies need processes and tools to identify sustainability risks in their SCs (Foerstl et al. 2010; Hartmann and Moeller 2014; Kashmanian and Moore 2014), particularly relying on exchange with their top tier suppliers (Leppelt et al. 2013; Sancha et al. 2016; Schoeggl et al. 2016b; Seuring and Gold 2013). One of the primary means for obtaining this sustainability information, necessary for a focal company’s SSCM, is through supplier sustainability assessment (Lee and Kashmanian 2013; Schoeggl et al. 2016a; Fraser et al. 2020b). Seuring and Gold (2013) note that more research in the direction of supply chain sustainability *performance* was needed. Dubey et al. (2017b) find that assessing suppliers’ sustainability performance is crucial for continuous improvement. Among other things it helps to quantify sustainability performance and thereby contributes to the successful implementation of SSCM (Das 2017). Mueller and Bessas (2017) found that of fourteen sector-based initiatives analysed, twelve of them involved focal companies employed a type of supplier sustainability assessment in the form of questionnaires and/or audits. Canzaniello et al. (2017) found that companies join industry associations (‘strategic alliances’) to improve the equivocality of supplier assessments and hence improve the ability to share supplier assessment data. Given the growth of SSCM literature, it is surprising that there is little literature that focuses on the ‘how to?’ of assessing suppliers’ sustainability. Furthermore, there is still a particular lack of research dealing with the operative and applied active assessment of suppliers’ sustainability practises.

### Supplier self-assessment in SSCM

As illustrated in Fig. 1, Dubey et al. propose that supplier assessments are tools within “performance assessment” and constitute one of 18 SSCM items (2017a, p. 339). We propose that this item warrants more academic attention. Questionnaires are a common example of how companies gather sustainability data and assess their suppliers’ sustainability performance (see, for example: BSR & UNGC 2015; Ecovadis 2017). Other tools for operative sustainability performance assessment include code of conduct monitoring, audits, certification, sustainable labelling, creating minimum standards, tracking and tracing systems and multi-tier transparency initiatives (Dubey et al. 2017a; Fraser et al. 2020a; Kashmanian and Moore 2014; Mueller and Bessas 2017).

Despite the lack of apparent academic focus on questionnaires as an integral tool of SSCM, they can be found around the world in all types of industries (BSR & UNGC 2015) and settings, ranging from the building and construction industries, universities and government departments to large manufacturers, food and beverage retailers and financial institutions. A number of industry initiatives, such as the automotive industry’s AIAG and DRIVE Sustainability, the electronic industry’s EICC^1^, the toy industry’s Ethical Toy Programme (IETP), or the chemistry industry’s Together for Sustainability, continue to further develop common and standardized sustainability questionnaires that are given to first tier suppliers^2^. However, widespread use of such tools does not preclude challenges involved with employing self-assessment as a tool, which is why we look at potential problems in self-assessment as applied to supplier sustainability management.

### Challenges in supplier self-assessment

Much has been written on the subject of questionnaire design, with research spanning half a century and continuing into the present (for example: Bradburn et al. 2004; Dalal and Hakel 2016; Jenkins and Dillman 2012; Krosnick 2018; Schwarz et al. 1991; Tourangeau 1984; Wright and Barnard 1975). In short, respondent’s reactions and responses to questionnaires are affected by: the language used, the questionnaire mode, the way the questions are constructed, the self-assessment process that the questionnaire is embedded in and the visual/spatial construction of the questionnaire (Jenkins and Dillman 2012). Despite much cognitive and organisational research into questionnaires, there remains much to be discovered and improved (Vannette and Krosnick 2018). It follows that SSCM should heed the lessons learned in other academic disciplines and pay particular attention to the tools used to gather sustainability information from the supply chain. One major problem that emerges from self-assessment is social desirability bias.

Walker and Jones (2012) note that despite much progress in the field of sustainable procurement, there are methodological challenges that apply to all corporate social responsibility-related fields (i.e. sustainable procurement, SSCM, green SCM). When respondents are asked to answer questions about sustainability, they “are often compelled to give a positive impression of their own and their organisation’s activities” (Walker et al. 2012, p. 202). This is not caused by explicit external pressure but rather due to social desirability bias, one of the main problems encountered when direct responses from individuals are sought on moral topics. Whilst van der Vaart and van Donk (2008) critically assess various subjective interpretations of supplier relationships and the dependence on individual assessment of supply chain integration, they do not address the issue of social desirability bias.

In other fields, such as social psychology and organisation behaviour, this bias and the inherent methodological limits of self-report questionnaires have been widely discussed (Dalal and Hakel 2016; Joinson 1999; Rolstad et al. 2011; Scherpenzeel and Saris 1997; Sjöström et al. 2009; Spector 1994), but this debate is barely found in operations research on inter-firm relations, let alone in SCM. One exception is the work of Roxas and Lindsay (2012), which addresses the issue of social desirability bias in small firms and the differences in ‘accurate’ sustainability reporting. They found that purely self-administered questionnaires led to less accurate reporting and overly positive self-assessment of sustainability. These results confirm the findings of Angus-Leppan et al. (2010) in which respondents were inclined to characterize beliefs and ideal states in an overly positive manner, hindering their ability to realistically disclose sustainability management practices. In all buyer-supplier interactions involving self-assessment questionnaires, individuals’ innate biases very probably have an impact on the resulting sustainability performance. Leppelt et al. (2013) take care to address these issues of misreporting and bias in the data for their research into business relationships and SSCM, but so far little work has been done on ensuring the same when designing sustainability self-assessments questionnaires for suppliers.

Beyond the risk of social desirability bias, we should consider the problem of supplier assessment fatigue (Ecovadis 2017; Jiang et al. 2013; Kashmanian and Moore 2014; Newitt 2012; Zamur et al. 2017; Fraser et al. 2020b). Focal companies have responded to the pressure to implement supplier sustainability assessments (BSR and UNGC, 2015). Thus their suppliers receive various sustainability questionnaires and requests for certification and audits, often receiving similar requests from multiple customers (Davies 2012; Ecovadis 2017). This can lead to supplier “fatigue” as supplier production facilities have to incur various compliance cost burdens (Newitt 2012; Fraser et al. 2020b). Initiatives such as Together for Sustainability and Drive Sustainability recognised this burden and identified it as a driver for standardisation (Drive Sustainability 2017; TFS—Together for Sustainability 2016). Jiang et al. (2013) took supplier survey fatigue as a starting point for their research into the challenges and opportunities for supplier disclosure to their customers in SSCM. Grewatsch and Kleindienst (2017) show how cognitive frames affect organizational capabilities, with a particular focus on corporate sustainability capabilities. Accordingly, similar to individuals, companies can take on cognitive identities that then affect the individuals within them (ibid), which could influence an individual’s responses. It follows that when individuals are responding to self-assessments on behalf of companies we must ask ourselves to what extent an individual is responding in a socially desirable manner and how this affects the company’s sustainability results in a SSCM context.

**Fig. 2:**
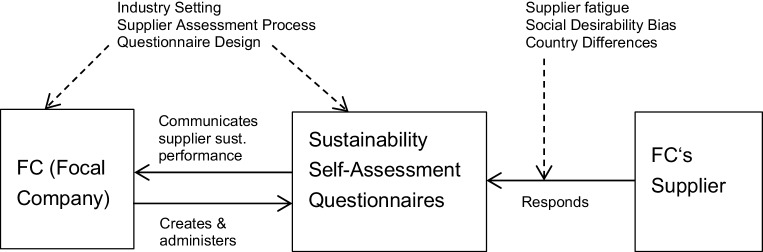
Research Model demonstrating process and potential variables affecting supplier responses

In the literature we find several potentially significant factors that could affect supplier assessments and self-assessment questionnaires, and importantly the results they produce: social desirability bias (and the supplying company’s cognitive frame), questionnaire design and process, supplier assessment fatigue, and the focal company’s institutional setting. We also assume that country differences would be a factor in performance variance (see, for example, Jia et al. 2018b). The proposed influencing factors and the relation of these factors to one another in the supplier sustainability assessment process can be seen in Fig. 2.

We have established a need for empirical analysis of supplier sustainability self-assessment questionnaires to investigate what the critical factors are and how they potentially influence supplier responses. In the following section we introduce the multiple-case study design and our abductive method, before describing two case studies focusing on supplier sustainability questionnaires that we conducted to address this research gap.

## Method and multiple-case study design

From the literature it emerges that supplier self-assessment questionnaires play an important role in SSCM but have received little critical academic attention. Hence, we decided to research two available cases of such self-assessment questionnaires and to compare and contrast them in terms of the processes associated with them and the results they generate. Yin (2018) notes that case study research, which seeks to understand complex phenomena in their real world contexts, is well suited to questions of inquiry that seek to understand ‘how’ and ‘why’ things occur. Our research involved an empirical inquiry into how suppliers respond to sustainability self-assessment questionnaires and why they might potentially respond in certain ways. Ultimately, the research design, involving multiples cases, allowed us to not only focus on specific variables and results but also the potential for cross-case synthesis. This synthesis involves uncovering within-case patterns and comparing these across the cases in order to determine literal replication (same or similar results in different cases) or theoretical replication (despite case similarities, divergence of outcomes due to predictable factors) (Yin 2018, pp. 174–194).

### Research method: design, approach and unit of analysis

We pursued our research based on a qualitative, abductive research design (Helmreich 2007; Ketokivi and Mantere 2010; Timmermans and Tavory 2012). After reviewing the extant literature, we began collecting and analysing our data. We then noted emerging themes and generated conceptualisations and then returned to the literature and repeated this abductive process in several iterations (Biggs 2011; Hahn and Ince 2016; Timmermans and Tavory 2012). In this manner the case studies can serve to improve extant knowledge with the insights gathered from the idiosyncrasies of the empirical context (Ketokivi and Choi 2014; Stuart et al. 2002). The organisational *context* for both case studies was a global automotive focal company “AFC” (henceforth referred to as AFC). The *unit of analysis* was the sustainability self-assessment questionnaire, administered to this company’s suppliers. The *subjects* of this research, AFC’s suppliers, responded to one or both sustainability questionnaire/s requested by AFC. Thus, our research involved a multiple case-study design wherein the same unit of analysis, the supplier sustainability questionnaire, formed the basis of two case studies (Yin 2018). As detailed in subsection 3.3. below, the questionnaires were similarly structured (indeed, experience gained with the first questionnaire informed the development of the second questionnaire) and fulfilled the same monitoring purposes as part of AFC’s sustainable supply chain activities. By analysing two cases involving a large amount of supplier response data gathered over a timeframe of five years, we aimed to identify meta-level potential causal factors that could lead to new understandings for academia and practice.

### Case studies: background and setting

Despite the widespread use of supplier sustainability questionnaires in practice and their central place in the canon of SSCM tools and processes (Lee and Kashmanian 2013; Mueller and Bessas 2017), there has been very little academic inquiry into these tools. Consequently, we explored this topic by analysing case data on two supplier sustainability questionnaires and assessment approaches administered by AFC. Whilst each case concerns a different questionnaire, both questionnaires fulfilled a very similar purpose as part of SSCM monitoring. Case A concerns the original, internally-developed supplier sustainability questionnaire (ISQ) and Case B refers to the more recent, industry-wide developed supplier self-assessment questionnaire (SAQ) on sustainability that AFC gathered from some of its suppliers.

#### CASE A: Internal Supplier Questionnaire (ISQ)

AFC began assessing its suppliers on sustainability in 2006. It created a supplier questionnaire to monitor its suppliers’ compliance with AFC’s sustainability requirements for business partners. The questionnaire was piloted in 2006 and developed over the ensuing years. In 2012, after years of manual processing, AFC began recording all supplier responses to the ISQ in a central database. Completion of the ISQ was mandatory for those suppliers concerned (defined as over a certain amount of turnover, high-risk, or as a measure/response to concrete suspicions of infringement) in the timeframe under consideration (2012–2017). The ISQ was technologically integrated into the central business partner platform and the completed ISQs could be checked by AFC’s buyers in the supplier database.

### Case A: ISQ design and integration in AFC’s processes

**Fig. 3:**
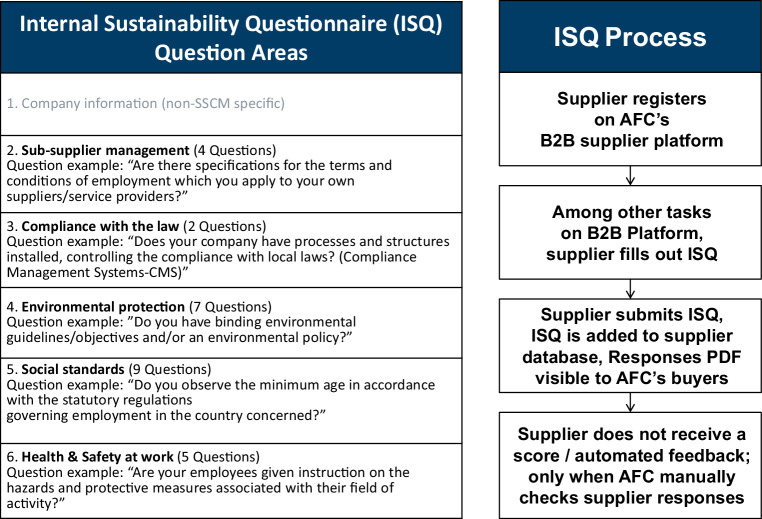
ISQ questionnaire design (sustainability areas & example questions) and ISQ process

The ISQ was designed to cover major areas of AFC’s sustainability requirements for suppliers. It is divided into five major sustainability areas, with each area containing between two and nine questions (see Fig. 3). All questions required a “yes” or “no” answer. The total score for each ISQ was generated by dividing the number of correct responses (points) by the total number of questions (24). All but one question in the ISQ, required a ‘yes’ in order to score a point. The ISQ process had been technologically integrated into supplier onboarding processes since 2012. The supplier received a task as part of this process and reminders to complete the ISQ. In certain cases, suppliers were contacted regarding their responses, which led, in some cases, to suppliers submitting updated ISQs.

### CASE B: European Automotive Industry Self-Assessment Questionnaire (SAQ):

In 2013, an Automotive Original Equipment Manufacturer (OEM) working group for sustainability in global supply chains was officially launched (CSR Europe 2013). Based on their respective experiences with sustainability questionnaires, combined with their suppliers’ feedback that suppliers were receiving too many individual supplier sustainability questionnaires from OEMs, the working group created a common Self-Assessment Questionnaire (SAQ) on sustainability for suppliers, which was publicly launched in April 2014 (CSR Europe 2014a; Drive Sustainability 2017). The SAQ built upon the Automotive Industry Guiding Principles to Enhance Sustainability Performance in the Supply Chain, which was co-launched by CSR Europe and the Automotive Industry Action Group (AIAG) in March 2014 (CSR Europe 2014b).

### Case B: SAQ 2.0 design and integration in AFC’s processes

**Fig. 4:**
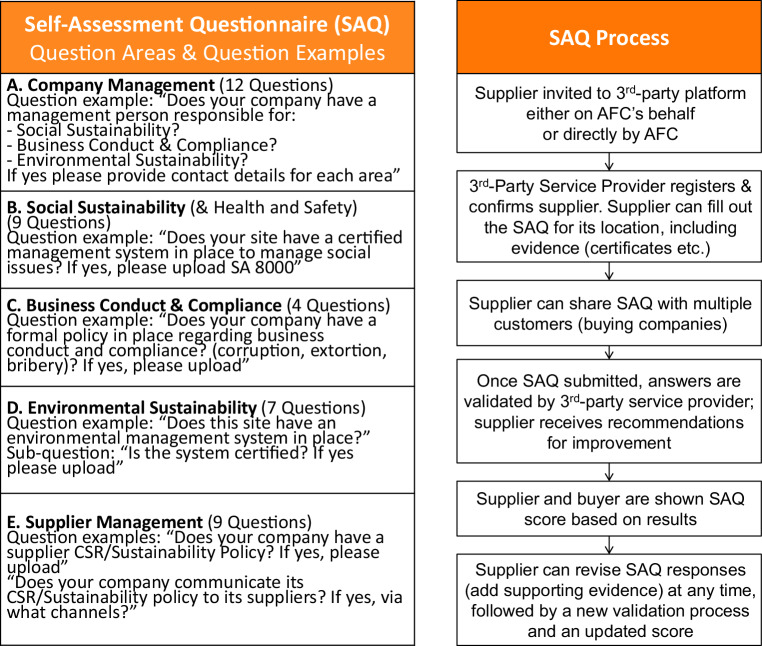
SAQ questionnaire design (sustainability areas & example questions) and SAQ process

The SAQ evolved from previous experience with supplier sustainability questionnaires and aimed to strike a balance between detailed questions addressing core suppliers’ sustainability practices without being overburdening. Thus questions did not just ask whether a supplier had capacity, a sustainability policy or a certified management system, or was undertaking certain measures, but also required evidence for these claims. To remove the processing burden from participating OEMs and to ensure that supplier data was not shared with other OEMs against a supplier’s will, the administration was undertaken by the third-party service provider, under the anti-trust oversight of CSR Europe. Each OEM could choose to what extent and how the SAQ was integrated into internal procurement processes and systems. AFC chose to participate in the SAQ sharing platform solution. Suppliers were invited (see Fig. 4) as part of campaigns run together with the service provider, individually or in waves organised by AFC. A supplier was asked to register their supply location on the platform, fill out contact and location details, and answer the sustainability questions. The supplier was then checked by the third-party platform provider. Often the responsible person for completing the SAQ had to gather information, certificates, links and other evidence in the process of filling out the online questionnaire^3^. Once completed, the supplier could choose which OEMs to share their SAQ with. Thereafter the SAQ was validated by the platform provider, to check if the attached evidence supported the responses. Finally, a percentage score was given based on the responses. To begin with, this score could only be seen by the OEMs with whom the supplier had chosen to share the SAQ; the supplier only saw a coloured ‘award’ (red, amber or green). As of early 2017 the suppliers could also see their percentage score.

AFC began inviting a small number of supplier locations in 2014 to test the SAQ. Increasingly, AFC began to onboard its most critical and production-essential suppliers. By 2017 AFC had plans to integrate the SAQ into its procurement processes and systems, so that buyers could see the SAQ results for potential and existing suppliers. The SAQ also informed AFC’s risk management and more suppliers were successively invited via strategic campaigns.

Apart from a small number of non-weighted questions and sub-questions, each ‘mandatory’ question resulted in an equal percentage contribution to the total score. Service provider suppliers that were not production site locations did not have to answer as many questions as production locations, but still received a score out of 100%. However, all suppliers invited to fill out the SAQ by AFC were suppliers of parts and components for production (not service providers). Importantly, at no point in the timeframe under consideration was the SAQ mandatory for suppliers. They were strongly encouraged by the procurement department, but by no means did all invitees register on the platform. Furthermore, not all of those who did register completed a SAQ and not all who completed it shared their SAQ with AFC.

### Methods: research procedure

Two members of our research team were able to interact with AFC at different times over a timeframe from 2012 until 2018. Therefore, the data collection, data extraction and data analysis were conducted in a collaborative manner, allowing the researchers to make observations over time that supported the raw data in the form of the suppliers’ questionnaires. Both case studies were carried out by the same researcher, who was based at the automotive focal company throughout the research, with access to both databases and all material required. The data informing both cases primarily consists of empirical data in the form of direct supplier questionnaire responses, as well as supporting participant observation carried out by the researcher who was entrusted with analysing both the questionnaire data itself as well as the processes of data collection.

#### CASE a: ISQ data collection

AFC’s ISQ database contained over 20,000 supplier sustainability responses. The dataset contains every response recorded in the timeframe from January 2012 until December 2017. Each supplier location’s responses were collated in the database. The data were extracted on a single day in January 2018, representing a snapshot of all collected responses up to the end of 2017. The database portrayed each supplier location’s responses to individual questions, grouped into sustainability areas, and also contained information on the country location. By means of a unique identifier duplication could be ruled out.

#### CASE b: SAQ data collection

The SAQ data was administered, processed and validated by the platform provider. Therefore, AFC’s supplier SAQ data were extracted from the service provider’s secure platform. Between 2014 and the end of 2017 there were 8093 of AFC’s supplier locations registered on the platform. The final SAQ dataset, forming the basis of Case B, involved 5431 completed SAQ responses. The other 2662 SAQs had not been completed and thus had no score for analysis. A supplier could return to their SAQ and update their responses, resulting in new validation and (potentially) a new score. Our dataset only contains the most recent score for each location as the final score. The database contained country location information and separated each supplier’s response to individual questions. By means of a unique identifier number SAQ duplication could be ruled out with high certainty. The platform provider undertook regular data quality checks to ensure supplier data was current.

#### Cross-case synthesis: comparison and analysis

We had the unique opportunity to access two large questionnaire datasets at a time when AFC was shifting its strategy towards more shared supplier assessment. This window of opportunity combined with personal access to AFC’s systems and processes meant that a thorough analysis of each case could be undertaken. By analysing total score distributions, and then further sorting response scores into sustainability areas as well as filtering total scores according to countries, we could synthesise the two cases and begin to compare the case data. Various findings arose and by applying abductive logic we proposed a number of variables for better explaining supplier responses to these SSCM tools.

## Results

In the following we present the results of the two questionnaires that constitute the unit of analysis of each cases study. The results depict the distribution of the overall supplier scores for each case, the average scores for sustainability areas in the questionnaires and the countries with the largest number of supplier locations and the average questionnaire scores at a country level.

### CASE A: Internal Sustainability Questionnaire (ISQ)

**Fig. 5:**
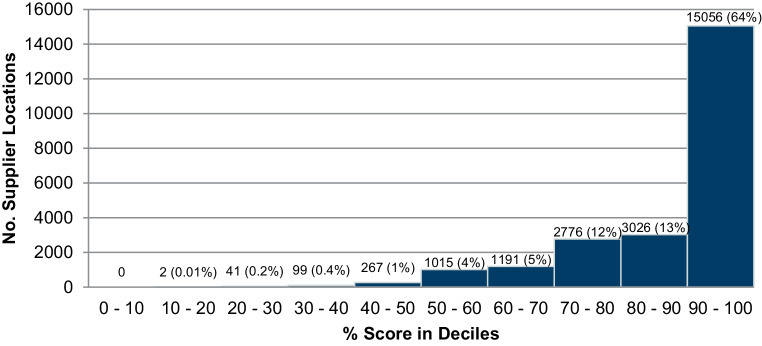
Supplier locations’ ISQ % Scores, distributed by score decile

In total there were 23,473 ISQ responses collected. If an ISQ was resubmitted, the latest response replaced the previous entry in the database, so that all responses were unique. 9224 locations scored a perfect score (24 out of 24 possible points), representing nearly 40% of all ISQs. Scores ranged from 17% to 100% but the *average score was 89%*. Fig. 5 depicts the frequency distribution of the supplier locations’ ISQ scores per decile. It is very evident that ISQ scores between 90% and 100% were the most frequent: the majority (64%) of all supplying locations achieved a sustainability score in this decile. Nearly 90% of ISQ scores were in the top three deciles. Conversely, less than two per cent of all supplier locations had scores of less than 50%.

The ISQ was divided into 5 sustainability areas. We analysed the average scores by sustainability area, which ranged from average scores of 79% for Environment to 96% for Social Standards (see Table 1). These high sustainability area scores are understandable considering the average score across all ISQs (89%) and that 40% of all ISQs collected had scores of 100%.

**Table 1:** Averages scores by ISQ sustainability area

Average Total ISQ Score	Sub-suppliers	Compliance	Environment	Social Standards	Safety & Health in workspace
89%	82%	94%	79%	96%	92%

ISQs were collected from supplier locations from 80 countries. Half of all ISQs came from supplier locations in Germany, wherein the average ISQ score was 86%. Overall, 80% of ISQs came from supplier locations based in just 10 countries. Table 2 shows that there was not much variation in average ISQ scores across countries^4^: Germany had the lowest, 86% and China the highest average score, 98%. We now turn to the results of the second questionnaire, the industry-wide self-assessment questionnaire, that constitutes Case B.

**Table 2:** ISQ distribution & average scores by top 10 countries

	Supplier Location Country	No. Supplier Locations	% of all ISQs	Average score %
1	Germany	12,331	52.5	86
2	Mexico	1110	4.7	92
3	Brazil	995	4.2	94
4	USA	915	3.9	90
5	Spain	811	3.5	95
6	Czech Republic	739	3.1	93
7	China	728	3.1	98
8	Italy	671	2.9	91
9	United Kingdom	527	2.2	90
10	Poland	510	2.2	93
	*Total Top 10*	*19,337*	*82*	*92*
	All suppliers ISQ	23,473	100	89

### CASE B: Sustainability self-Assessment Questionnaire (SAQ)

**Fig. 6:**
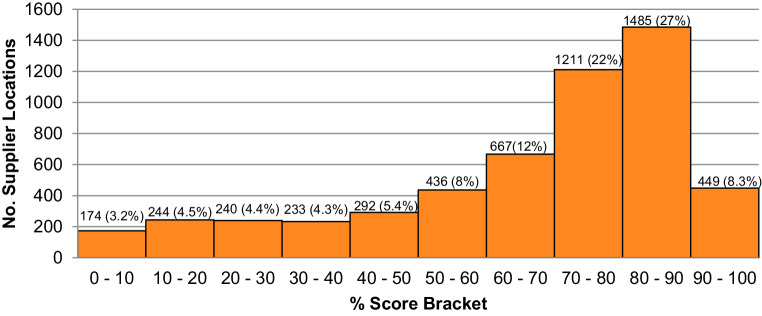
Distribution of supplier locations’ SAQ % scores, by score decile

The results considered in this research are the score that each supplier location last achieved. Only four locations achieved a perfect score (100%), whereas 90 supplier locations submitted a SAQ but received a score of 0%. Thus, the range of SAQ scores was from zero to 100%, with an average score of 65%. The distribution of SAQ scores per decile can be seen in Fig. 6.

We observe that over half of all supplier locations (58%) in the sample achieved a SAQ score in the top three score deciles. Conversely, 42% of all supplier locations got SAQ scores in the lower seven deciles, with more suppliers achieving scores between 50 and 70% and an otherwise fairly even distribution across the score deciles zero to 50%.

The SAQ was divided into five sustainability areas. We analysed the average scores by sustainability area across all supplier locations, which ranged from an average score of 52% for social sustainability to 72% for environmental sustainability (see Table 3).

**Table 3:** Average SAQ scores by sustainability area

Average Total SAQ Score	Supplier Management	Business Conduct and Compliance	Environmental Sustainability	Social Sustainability	Company Management
65%	54%	67%	72%	52%	70%

These sustainability area scores indicate that AFC’s suppliers achieved higher scores in the areas of environmental sustainability, company management and business conduct and compliance, but only achieved mediocre scores for social sustainability and supplier sustainability management.

SAQ data was collected from supplier locations in 70 different countries. Germany-based supplier locations constituted over a third of all responses and the top ten supplier location countries accounted for over two-thirds (72%) of all SAQ responses in total (see Table 4). Among the top ten countries, average SAQ scores ranged from 60% (United Kingdom) to 73% in Mexico, indicating low average score variance among countries. When considering the top 20 countries, it is interesting to see that the highest average score was found among suppliers in Romania (77%), whereas the lowest average SAQ country score was in Denmark^5^. In the following we discuss these results and findings from each case, and then synthesise cross-case patterns of similarity and divergence.

**Table 4:** SAQ distribution & average scores for top 20 countries

	Supplier Location Country	No. Supplier Locations	% of all SAQs	Average score %
1	Germany	1927	35.5	62
2	Sweden	317	5.8	62
3	Mexico	268	4.9	73
4	Italy	235	4.3	61
5	Czech Republic	231	4.3	70
6	Spain	220	4.1	67
7	Poland	196	3.6	72
8	Brazil	173	3.2	65
9	United Kingdom	164	3.0	60
10	France	155	2.9	70
	*Total Top 10*	*3886*	*72*	*66*
11	China	136	2.5	73
12	Hungary	124	2.3	70
13	Austria	115	2.1	69
14	United States	114	2.1	72
15	India	106	2.0	64
16	Slovakia	103	1.9	72
17	Turkey	84	1.5	68
18	Romania	75	1.4	77
19	Netherlands	66	1.2	56
20	Denmark	52	1.0	51
	*Total/Average Top 20*	*4861*	*90*	*66.7*
	All Suppliers’ SAQs	5431	100	65

## Discussion

The academic benefit of conducting multiple-case study research lies in finding like cases, with the same unit of analysis (in this case: sustainability questionnaires for suppliers). These can either predict similar findings across multiple-cases, resulting in ‘literal replication’, or point to like cases that predict contrasting results but for anticipatable reasons, so-called ‘theoretical replication’ (Yin 2018, p. 57). If either of the results for a particular questionnaire presented above were to be analysed alone, the question might arise, how realistic are these results? Whilst analysing the results of the two cases it became apparent that the meta-results differed in important ways. Although different questionnaires, due to their similar content and purpose a meta-level comparison of the amassed data provided us with insights into supplier sustainability questionnaires as a SSCM tool. Furthermore, both questionnaires served as monitors of suppliers’ (reported) sustainability compliance and an assessment of supplier sustainability practices. In general, we found cause for scepticism about how representative the ISQ results are of sustainability performance and that the SAQ is probably a more accurate SSCM tool. In the following, we present our cross-case synthesised findings.

### Findings: distribution of average supplier scores

As presented in 4.1 and 4.2, it is worth recalling and synthesising the score distributions of the two questionnaires to make the major finding of our research clear. The SAQ returned a visibly more diverse set of supplier responses/scores than the ISQ. The ISQ responses resulted in very high scores (average 89%) and accordingly a high concentration of high-scores (nearly 90% of all supplier locations responses returned scores between 70% and 100%, recall Fig. 5). The extremely high average ISQ scores and the corresponding lack of differentiation among suppliers’ performance meant that deeper analysis of the ISQ results and drawing meaningful conclusions were rendered superfluous. The SAQ responses returned greater variation in scores. The average score was lower (65%) with 42% of supplier responses generating scores between zero and 70%. The SAQ results demonstrated a somewhat top-heavy distribution of scores, with nearly half of all supplier locations achieving scores between 70% and 90% (recall Fig. 6). Still, due to the broader spread of scores (contrast Figs. 5 and 6), it was possible to differentiate more among supplier responses, also when considering other aspects such as questionnaire sustainability area and country results.

### Findings: sustainability area scores

Both questionnaires contained different sustainability areas, meaning that supplier location responses could be looked at thematically. Table 1 (Sect. 4.1.4) and Table 3 (Sect. 4.2.4) depict the average scores drawn from the responses based on each sustainability section in the respective questionnaires. The ISQ returned slightly less varied responses when considering average scores by sustainability area and much higher average area scores in total. The highest average ISQ section scores were for social standards (96%) and the lowest for environmental protection (79%). However, given justifiable scepticism about ISQ results in general, we focus on SAQ section results. SAQ sustainability section average scores were significantly lower and ranged from 52% for social sustainability to 72% for environmental sustainability^6^.

The SAQ sustainability section results coincide with general literature findings, namely that it is harder to quantify social sustainability and harder for firms to measure and improve performance in this area (Beske et al. 2014; Dubey et al. 2017a; Fish 2015). Thus, suppliers were more likely to achieve higher environmental sustainability scores in the SAQ thanks to ISO 14001 certification and certified management systems etc. Unlike the ISQ, the SAQ points not only to social sustainability as a SSCM area deserving continued attention (Hutchins and Sutherland 2008; Ferri and Pedrini 2018) but also to sub-supplier sustainability (Fraser et al. 2020). These findings correspond well with current problems for practitioners around achieving transparency in SCs (BSR and UNGC 2015; Schwarzkopf et al. 2018), addressing SSCM risks in raw materials SCs and how to pass on requirements beyond tier one suppliers (BSR and UNGC 2015; Drive Sustainability 2017; Sauer and Seuring 2017; Jia et al. 2018a). The SAQ responses demonstrate interesting and differentiated findings about supplier sustainability practices.

### Finding: score differences based on location

ISQ responses were gathered from supplying locations in 80 countries and SAQ responses from 70 different countries. Eight of the top ten countries were the same for ISQ and SAQ responses. The top ten supplier locations countries constituted over 80% of all ISQ responses and over 70% of all SAQ responses. ISQ country results were not diverse, due to the limited range and very high average-scores. All countries from which we analysed three or more ISQ responses achieved an average score of *over* 85%. When we consider countries, for which we received three or more SAQs, the country average SAQ scores range from 36% to 85% indicating much more diverse responses and corresponding sustainability performance. Our results included an over-representation of Germany-based supplier locations, both for the ISQ and SAQ.

ISQ results, while demonstrating generally high scores, also demonstrated some counter-intuitive results. Supplier locations based in OECD countries such as Germany (86%) and the United Kingdom (90%) would normally achieve higher average sustainability scores than those supplier locations based in countries such as China (98%) and Brazil (94%). The country responses for countries 10–20 continued to challenge common-sense wisdom: Russia and South Africa (92%) and India (93%) achieved higher average ISQ sustainability scores than supplier locations based in Austria (89%), the USA (90%) and Italy (91%). Thus, ISQ results can be questioned as not being indicative or representative of country sustainability practices and performance^7^.

SAQ responses, however, also returned country results that were contrary to our expectations. For example: Mexico (73%), China (73%) and Brazil (65%) achieved higher average SAQ scores than Germany (62%), Sweden (62%) and the United Kingdom (60%)^8^. As described, these suppliers had to upload evidence to support their SAQ responses, which means that there is little basis for questioning the accuracy of the individual location scores, and therefore the aggregated scores. However, self-selection bias could account for some of the unexpected higher country-score averages. The SAQ was not mandatory for AFC’s supplying locations in the timeframe under examination. Several thousand supplying locations had registered on the platform between 2014 and 2017 but did not complete or share their SAQ with AFC. Some suppliers began to fill out the SAQ and then stopped, possibly realising that completing it would probably return a low score. Other suppliers, upon realising that the SAQ was not mandatory, might not have wanted to disclose their porous sustainability practices. One can similarly surmise that suppliers who were confident of achieving higher scores would be more likely to complete and share a SAQ. German and Swedish suppliers were prioritised for various reasons by AFC. Possibly these country results are more statistically representative in general. These supplying locations might have been more likely, due to historic and strategic partnerships, to feel compelled to complete and share their SAQ with their customer AFC.

The country responses showed the SAQ responses are probably *not* representative of general sustainability risks indicated by the respective social economic development level of those countries, prominent examples being China, Mexico, Turkey and Romania outperforming Denmark, Germany and Sweden. With nearly 2000 data-points, the results for German suppliers could possibly be considered representative, but all other countries (from which we gathered less than 320 SAQs each) cannot plausibly be considered representative of sustainability performance at country level.

## Conclusions, limitations and relevance for SSCM

We began our research asking ourselves what the salient differences are between two types of suppliers’ sustainability self-assessment questionnaires. With access to a large amount of data from two different types of questionnaires (designed to fulfil the *same* SSCM *purpose* and constituting the *same type of tool*), we wondered how suppliers might respond to them, how these responses might differ, and if so, why this might occur. We proposed that responses to self-assessment questionnaires could be influenced by: supplier assessment fatigue, a supplier employee’s social desirability bias (and the supplying company’s cognitive frame), questionnaire design and how it is embedded in company processes, the focal company’s institutional setting and potentially by country differences. We conclude our article with major conclusions resulting from our cross-case comparison and findings before illuminating limitations to our research and making recommendations for future research. Finally, we highlight the relevance of this research for SSCM academics and practitioners.

### Main conclusions on self-assessment questionnaires for SSCM

In response to a demonstrated research gap and directed by our two research questions, this research demonstrated how such supplier sustainability self-assessment tools can differ, both in terms of their questionnaire structure and the institutional setting and company processes that they necessitate. It then sought to explain why supplier responses might differ, depending on the supplier self-assessment tool. In the following we expound on the role and importance of questionnaire design, assessment process and institutional setting, concluding that more diverse, realistic supplier assessment tools are essential for SSCM.

#### Addressing SDB and cognitive frame: questionnaire design & process

We found that both questionnaire design and the process seemed to influence the responses and could potentially limit social desirability bias and address negative cognitive framing. In other disciplines much attention has been paid to questionnaires (such as the order of questions, leading vs. open questions etc.) and their design (Bradburn et al. 2004; Dalal and Hakel 2016; Jenkins and Dillman 2012; Tourangeau 1984). In our case studies we clearly observed that the ISQ’s ‘leading questions’ (that were noticeably looking for one type of socially desirable response) were overwhelmingly responded to in the expected manner. Apart from one question that required a “no” answer, it could be inferred that those filling out the ISQ were aware that “yes” was the desired response. SSCM tools are designed to assess suppliers, but those responding to such questionnaires are still individuals, who are subject to psychological tendencies such as social desirability bias. The ISQ can thus be defined as a form of self-report.

By contrast, the SAQ can only be defined as semi-self-report due to the validation that took place after responding. Suppliers were aware of the process and design of the questionnaire and that their answers would be validated. The final score, therefore, did not necessarily represent the initial self-reported responses, as those responses that could not be supported had to be revised. SSCM questionnaires and other assessment tools and methods must be designed with social desirability bias and supplier assessment fatigue in mind. Our cases demonstrated how both questionnaire design and assessment process can potentially reduce the tendency to respond in a socially desirable manner. Standardising SSCM tools and enabling results sharing among an industry can reduce the fatigue of those suppliers responding to multiple buying focal companies. Moreover, we observed that the SAQ and the supplier results served as the basis for dialogue between AFC and its suppliers for further supplier development. As the SAQ could be revisited by the supplier, it enabled continued supplier sustainability development and potentially created a different impression on those completing the questionnaire.

#### Addressing supplier assessment fatigue: SSCM processes & institutional setting

AFC (presumably like many focal companies) began their SSCM journey by developing their own solutions to new requirements and reacting to emerging challenges and sustainability expectations. In this context, larger companies might initially develop their own supplier sustainability assessment tools. However, given overlapping supply chains, particularly when it comes to multi-tier, globally dispersed supplier networks (Schoeggl et al. 2016a), large tier‑1 suppliers probably receive numerous and similar sustainability questionnaires from their (focal company) customers. If not, a supplier location may wonder what the purpose of such a questionnaire is and may not have the competencies or human resources easily available to respond to the questionnaire^9^. In both cases the questionnaire may be regarded as an annoying ‘must do’ that is essentially about ticking boxes for compliance. If the supplier has the impression that the questionnaire is a one-off matter, along the lines of “are you ethical—please tick yes or no” (Crane 1999), then much less importance will probably be attached to understanding the topics and making sure that policies, process and competencies are in place to ensure more sustainable practices. The responses made in the ISQ were self-reported and only checked through manual intervention by AFC on a case-by-case basis. While we could not gather exact data from suppliers on how many self-assessments they had undertaken for different focal companies, we generated SSCM process findings that could point to supplier assessment fatigue as an explanatory factor. A potential example of this is the surprising number of suppliers (over 250) who responded “yes” to an ISQ question which asked whether involuntary labour had been used. This anomaly was discovered by AFC and suppliers were confronted about their responses. Then a so-called ad-hoc case was opened, and suppliers received a list of measures that had to be addressed as part of a monitored due diligence process. Suppliers explained that their response must have been a mistake. We infer that the ISQ was possibly seen as a tick-the-box measure that was necessary to proceed with registration on the business partner portal. If suppliers believed that no follow up would occur, then they may have rushed through the questionnaire, ticking ‘yes’ without slowing down to understand the questions.

On the other hand, if the supplier understands the questionnaire to represent an entire industry’s sustainability expectations, and that multiple buyers could be analysing the results of the questionnaire, the importance attached to the questionnaire could be much higher. By contrast, the SAQ process may have given more of an impression of supplier development, rather than a quick, one-off compliance check. This is because once suppliers completed their SAQ, the SAQ platform and process generates an automated catalogue of recommended improvements (based on the gaps stemming from the suppliers’ responses). This allows for continued interaction between suppliers and focal companies across the industry, to address the gaps, risks and issues identified (Foerstl et al. 2015). Whilst in some cases suppliers reported that the SAQ was first filled out by an administrative person in the company with little knowledge of sustainability practices, once feedback came from buyers regarding the low score, the responses were corrected, and the performance improved. CSR Europe and the SAQ platform service provider both confirmed that numerous SAQs were being shared with up to five focal companies. Furthermore, conversations with individual suppliers demonstrated that they were glad that they only had to fill out one SAQ (compared to earlier years) and for the option to share it with multiple buying focal companies.

We therefore conclude that SSCM tools, particularly supplier assessments and questionnaires, must make better use of institutional (and industry) settings, to maximise standardisation and the perceived importance of the tool, and thereby its potential sustainability impact. One way to achieve this is for industry players to collaborate and cooperate, agreeing on common sustainability standards and the tools to measure compliance with these standards (Mueller and Bessas 2017). Suppliers, like focal companies, have limited resources and more standardisation reduces their burden while simultaneously making it clear that the tool is to be taken seriously.

#### Differentiation in SSCM responses (sustainability areas & country performance) desirable

Our research demonstrated that the aspects detailed above (industry setting, assessment tool standardisation, questionnaire design and process integration) can lead to more differentiated supplier responses. We conclude that more differentiation among supplier sustainability responses is of much more utility for SSCM. If all scores are the same, how should companies make sustainability-risk based decisions, prioritise supplier development, and select sites for more in-depth monitoring such as sustainability audits? Companies around the world are at very different stages when it comes to sensitisation for sustainability and the place it may have in their company (procurement) strategies. If the SSCM assessment tools return a homogeneous response landscape, then one must question the design of these tools and processes surrounding them. We found that more differentiation (such as in the SAQ results) allowed for meaningful further analysis of sub-areas of the questionnaire, directing AFC’s attention to sub-supplier management and social sustainability as areas requiring further development. One plausible explanation for the higher scores in some areas, is that SAQs were resubmitted by suppliers, often after being engaged by different OEMs at different times (including AFC), potentially regarding certain topics (for example energy management), to improve their SAQ performance in this area. As well as highlighting differentiated supplier performance and responses in regard to sustainability areas, our findings also covered thousands of suppliers in different country settings.

Country (or regional) differences are a continuing topic in SSCM, as their socio-economic conditions are a major factor in supply chain networks (Beske et al. 2008; Locke 2013). However, our results showed the need for caution. As depicted in 5.1.3, our findings could not be considered representative for most of the countries. But this raises questions about the extent to which questionnaires are able to generate responses that can be generalised to the country level, and whether country-based risk factors can be transferred to individual suppliers. Both a well-run factory, with good environmental social and environmental performance in a developing country, as well as a supplier in a highly developed country, barely conforming with minimal legal requirements (or even acting in contravention of them) are conceivable. Many focal companies have conducted country risk analyses, so it is plausible that sustainability trainings for suppliers in such countries partially explain the surprisingly high average SAQ scores for certain countries.

The two case studies help confirm our initial propositions that supplier sustainability questionnaire responses would be affected by social desirability bias, questionnaire design and process, supplier fatigue, and the focal company’s institutional setting. In addition, they help confirm that similar questionnaires can generate very differing responses. A self-assessment questionnaire that undergoes validation and whose results can be shared with multiple focal companies elicited responses that (with a higher degree of probability) are more realistic. If SSCM tools generate favourable responses, one must either conclude that the supplier locations were all very sustainable, or that the questionnaire did not fulfil its role as a tool to accurately capture suppliers’ sustainability practices. Such a concentration of positive responses renders any analysis nearly useless, as one cannot differentiate and determine which responses genuinely represent ‘good’ sustainability practises and which responded in a socially desirable manner. The SAQ produced more balanced results, that probably more accurately depicts sustainability practices and provided AFC a basis for further SSCM actions and decisions.

### Limitations and recommendations for further research

There were several limitations that potentially affected our research, one methodological limitation and a number relating to the questionnaire processes and the supplier sampling. We recognise the methodological limitations of comparing two different tools. However, various case study research has involved comparing differing interventions (policy, management approaches, systems and process) to address the same problem across cases (Yin 1989, 2018; Eisenhardt 1991; Cavaye 1996). In this vein, we argue that the two different questionnaires are comparable, because they were designed to fulfil the same task—monitoring supplier conformance with sustainability requirements and documenting their activities.

Regarding the questionnaire process, one limitation may have been the degree of obligation on suppliers to complete the two questionnaires. The ISQ was mandatory for suppliers concerned and was an integral part of AFC’s supplier onboarding process. The SAQ was never strictly mandatory in the timeframe under consideration (although the preferred tool by 2017) and thus if suppliers refused to complete or share the SAQ with AFC, there were no hard/straightforward consequences. As mentioned in 5.1.3, the SAQ responses probably reflect a degree of positive self-selection bias, as those suppliers with better performance were more likely to share their SAQs. However, assuming this bias had a significant effect, it would mean that more representative SAQ results would then on average be even lower, with those supplier locations ‘required’ to fill out the SAQ bringing down the average score.

A further limitation relates to the supplier country differences and potential cultural differences. A third of all SAQs and over half of all ISQs collected came from supplier locations in Germany. Interestingly, in both cases the German average scores were not as high as expected, particularly compared to other countries. With over 12,000 ISQs and nearly 2000 SAQs these country samples represented two large datasets for Germany-based suppliers and yet the average scores were significantly different (ISQ: 86%; SAQ: 62%). Due to the unreliability about the accuracy of the ISQ scores it would be worth focussing our attention on the SAQ scores. Unfortunately, the next largest country samples only involved a few hundred suppliers and thus cross-country comparisons are not fruitful. AsZamur et al. (2017) have recently shown in their research, there is preliminary support for their hypotheses that cultural and institutional background can influence the perception of suppliers’ managers of their customers’ sustainability requirements and the probability that they will be motivated to comply with them. As part of our research to manage and gather supplier’s responses, it also became clear that language difficulties meant that some suppliers understood that “yes” was the desired response, but not necessarily the content of the questions. Another important, potentially limiting factor on our results was company size. The SAQ requires formalised certifications (e.g. ISO 14001) and internationally certified management systems that are complex and expensive. For smaller suppliers such ‘sustainability investments’ represent a larger personnel and financial burden. Another limitation may be the timing of our research, as the SAQ (a tool employed by DRIVE sustainability) is finding increasing usage both within AFC and across the group of participating companies.

Finally, this research postulates probable explanations for suppliers’ responses to an SSCM self-assessment tool. These conclusions are based on thorough, qualitative analysis of a large number of questionnaire responses and observations of the management of the questionnaires over a five-year period at AFC. However, the research did not follow-up with suppliers to ask them for explanations of their responses. Future research should focus on suppliers’ perspectives on such SSCM assessment tools in order to make the processes and tools even more effective in terms of improving SSCM transparency and performance.

### Relevance of this research for SSCM

Our research aimed to critically examine an under-researched and yet centrally, operationally important component of established SSCM. By analysing two cases of sustainability self-assessment questionnaires, we could show that the setting in which such a tool is placed is important. Companies are well advised to consider psychological and organisational behaviour aspects surrounding the design of questionnaires before using them blindly as instruments that should inform SSCM strategy. Standardised questionnaire processes (at industry level, for example) reduce the burden on suppliers and potentially increase the chance of accurate results, as the supplier anticipates the validation and importance of their responses. If supplier sustainability assessment tools have a higher probability of returning more realistic and differentiated responses, then more reliable sustainability data can be generated. This more representative supplier data can lead to more focussed supply chain sustainability strategies and improved supply chain risk management, based on real data rather than estimations. The unexpected emergence of the Covid-19 pandemic and the serious disruptions it caused to global supply chains brought wider public attention to the vulnerability and centrality of SCs to our global economy. Sustainable supply chain management can continue to gain in significance through these recent developments as companies and consumers try to better understand where their products come from and how to make supply chains more resilient. Well designed and implemented SAQs, such as those case-studied in this research, could expand their scope, to include further aspects to better prepare for future pandemics and contribute to more sustainable supply chains.

Future research on SSCM assessment could focus more on country comparisons of sustainability performance, changes in supplier sustainability performance over time, cultural differences regarding sustainability disclosure, and testing the correlations of sustainability self-assessment questionnaire results with sustainability audit results. Given the increased interest in sharing assessment data among SC actors, research should also be directed towards how inter-industry assessment data can be exchanged (e.g. between the mining extraction sector, the automobile industry and the chemistry industry). Then companies can surpass questionnaires of the ilk “Are you ethical? Please tick yes or no” (Crane 1999).

## Appendix

**Table 5:** Side-by-side comparison of the two questionnaires, matched by relating sustainability area

Internal Sustainability Questionnaire (ISQ)	Self-Assessment Questionnaire (SAQ)
**1. Company Information**	**A. Company Management**
(Not part of ISQ—general company questions asked elsewhere)	*1. Does your company have a management person responsible for:* 1a. Social Sustainability ***** 1b. Business Conduct & Compliance ***** 1c. Environmental Sustainability ***** *2. Does your company publish a corporate social responsibility report/sustainability report?* ***** 2a. Is your most recent report 3rd party assured? ***** 2b. Are the operations of all your company sites included in that report? *3. Does your company have a Code of Conduct in place?* ******* 3a. Is the Code of Conduct enforced at this site? *4. Do you organise training sessions to enhance the understanding of Corporate Social Responsibility/Sustainability at your site?* 4a. On which of the following topics do you organise training sessions: – Code of Conduct – Social Issues – Anti-Corruption & Ethics – Health & Safety – Environmental Management *5. Have employees from your site participated in external Corporate Social Responsibility/Sustainability training?* 5a. Who organised the training: – An OEM ***** – The Automotive Industry Action Group ***** – European Working Group on SC Sust. ***** – Other *6. Does your company participate in any voluntary CSR/sustainability initiatives?* *******
**2 Sub-suppliers** 2.1 Does your company have its own suppliers/service providers 2.2 Are there specifications for environmental protection which you apply to your own suppliers/service providers? 2.3 Are there specifications for the terms and conditions of employment which you apply to your own suppliers/service providers? 2.4 Are there specifications on health and safety issues which you apply to your own suppliers/service providers?	**E Supplier Management** *20. Does your company have a supplier CSR/ Sustainability Policy?* ******* 20a. Which areas are covered by this policy? – Respect for human rights – No forced or compulsory labour – No human trafficking – No child labour – Working conditions – Remuneration – Non-discrimination – Freedom of association – Collective bargaining – Anti-corruption and bribery – Healthy and safety – Environment 20b. Which supplier category is covered by your CSR/Sustainability policy? – Direct procurement suppliers – Indirect procurement suppliers *21. Does your company communicate its CSR/Sustainability policy to its suppliers?* 21a How is the supplier CSR/Sustainability policy communicated? – During supply meetings – In contractual terms – Brochures, magazines, newsletter, webpage *22. Which processes do you have in place to ensure that your Supplier Sustainability Policy is efficiently implemented by your suppliers?* ******* – Self-assessment questionnaire – Audits conducted by the company – Supply meetings – Audits conducted by an external 3rd party auditor – Other (please specify) – None
**3 Compliance with the Law** 3.1 Does your company have processes and structures installed, controllingthe compliance with local laws? (Compliance Management Systems-CMS-) 3.2 When will you have installed a Compliance Management System-CMS?	**C Business Conduct and Compliance** *13. Does your company have a formal policy in place regarding business conduct and compliance? (corruption, extortion, bribery)* ******* 13a. Are the following areas covered by this policy or the related processes & procedures? – Corruption, including extortion – Bribery *14. Does your company have a formal policy in place regarding Competition Law Compliance?* ******* 14a. Does this site have a documented business conduct & ethics management system in place? *****
**4 Environmental Protection** 4.1 Creation and application of environmental management system 4.1.1 Have you set up a certified environmental management system? 4.1.2 If yes → Stop; If no → following questions: 4.1.3 Is an environmental management system without certification in use? 4.1.4 Does your organisation have a contact person with responsibility for environmental protection? 4.1.5 Do you have binding environmental guidelines/objectives and/or an environmental policy? 4.1.6 Did you define responsibilities in respect of environmental protections of your company? 4.1.7 Does a record exist of compliance with statutory and company regulations on environment protection? 4.1.8 Are your employees informed and qualified in respect of environmental protection	**D Environmental Sustainability** *15. Does your company have a formal environmental policy, which includes a commitment to legal compliance, continuous measurement andcontinuous improvements in environmental performance?* ******* 15a. Are the following areas covered by this policy or the related processes and working procedures? – Energy consumption – Water Usage – Air Emissions – Waste Management – Restricted substances & chemical handling – Other areas, please specifiy 15b. Does your company have annual objectives and activities accordingly in the areas covered by your environmental policy? ***** *16. Does this site have an environmental management system in place?* ******* 16a. Is the system certified? ***** If no → 16aa. Have internal environmental audits been conducted at this site? ***** *17. Do other production sites/locations have a certified environmental management system?* ******* *18. Does your facility use restricted substances or chemicals for your production? If yes → 18a.* 18a. Does your facility have work procedures to manage the use of restricted substances and chemicals? ***** 18b. Has the management system used for managing the usage of restricted substances or chemicals been certified? ***** *19. Do you upload your material data to the International Material Data System (IMDS)?*
**5 Social Standards** 5.1 Contact Person 5.1.1 Does your organisation have a contact person that is responsible for social standards? 5.2 Freedom of association 5.2.1 Do you grant your employees the right to form and/or join trade unions or employee-elected representative bodies in accordance with national statutory regulations? 5.2.2 Where this right is limited by local laws, are there alternative forms of co-determination for employees in your organisation 5.3 Discirmination 5.3.1 Do you guarantee equal opportunity and treatment—regardless of ethnic origin, skin colour, gender, religion, nationality, sexual orientation, social origin or political views as long as these views are based on the democratic principle and tolerance towards those who hold different views? 5.4 Forced labour/child labour 5.4.1 Do you use forced or compulsory labour, or involuntary work performed bydetainees? 5.4.2 Do you observe the minimum age in accordance with the statuatory regulations governing employment in the country concerned? 5.5 Remuneration 5.5.1 Are the remuneration paid and benefits provided at least equivalent to the national standard or minimum standard of the relevant national sector? 5.6 Working hours 5.6.1 Are the working hours at least in line with the national statutory guidelines or standards of the relevant national sector? 5.7 Employee information 5.7.1 Are your employees informed and qualified in respect of social standards?	**B Social Sustainability** *7. For which of the following social issues does your company have a policy in place?* ******* **–** Respect for human rights – No forced or compulsory labour – No human trafficking – No child labour – Working conditions – Wages & Benefits – Non-discrimination – Freedom of association – Collective bargaining *8. Does your site have a certified management system in place to manage the above mentioned social issues?* ******* – No, on none of the above – Yes (please upload SA8000 Social Management System) ***** – No, but have internal documented procedures and policies ***** *9. Have social audits/assessments been conducted at this site?* ******* – No – Yes, internal audits – Yes, external 3rd party audits *****
**6 Health and Safety at Work** 6.1 Are health and safety duties and responsibilities clearly regulated in your organisation? 6.2 Are hazards evaluated in your organisation, and are the resulting protective measures developed and implemented and is their effectiveness monitored? 6.3 Are precautionary medical examinations carried out in your organisation? 6.4 Does your company have an internally organised first aid facility? 6.5 Are your employees given instruction on the hazards and protective measures associated with their field of activity?	*10. Does your company have a written health & safety policy in place, which complies with industry, national and international standards? ** 10a. Have specific activities on health & safety been organised at this site during the last year? ***** *11. Does this site have a health & safety management system in place?* ******* 11a. Is the system certified? ***** If no → 11aa. Have internal H&S audits been conducted at this site? ***** *12. Do other production sites/locations have a certified health and safety management system?* *******
	**F Special Areas** *23. Is the 3TG (tantalum, tin, gold or tungsten) necessary to the production of your company’s products and contained in the finished product that your company manufactures or contracts to manufacture?*

## Footnotes

1 Since October 2017 rebranded as the Responsible Business Alliance: http://www.responsiblebusiness.org/.

2 Also more online or platform service providers are responding to the demand for supply chain sustainability assessment, i.e. Sedex (www.sedexglobal.com), Ecovadis (www.ecovadis.com) and NQC Ltd. (https://auto.nqc.com/).

3 The SAQ could be filled out, if necessary over an extended period of time, such that responses already given could be saved to work on later.

4 The next ten countries only accounted for 11% of all ISQ responses and had an average ISQ score of 92%, with average scores ranging from 89% (Austria) to 94% (Slovakia, Portugal and Sweden).

5 Also, unexpectedly, China and Turkey both registered country average scores of 73%; higher than the averages for all western-European countries.

6 Interestingly, the highest average section score for the ISQ (social standards) was the lowest average scoring section in the SAQ (52%). Conversely the lowest ISQ section average scores were for environmental protection (79%), whereas this was the section with the highest average scores (72%) for the SAQ.

7 For example, supplier locations based in developing countries like Iran and Botswana (score 100%), Philippines (99%), Indonesia (98%), Thailand (97%), Tunisia and Morocco (96%) and India (93%) paint a high-scoring picture for these countries when looking at ISQ responses.

8 Furthermore, countries such as the United Arab Emirates (81%), Timor-Leste (74%), Nicaragua (79%), the Philippines (85%) and Indonesia (83%) all demonstrated perhaps unexpectedly high SAQ average scores, but the sample size for these countries was very small (one to three supplier locations) and thus should not be considered representative for general sustainability performance in these countries.

9 Indeed, the researchers were confronted time and again with suppliers who genuinely wondered why they ‘had’ to fill out such questionnaires and what it might be good for.
